# Construction and validation of an oxidative-stress-related risk model for predicting the prognosis of osteosarcoma

**DOI:** 10.18632/aging.204764

**Published:** 2023-06-02

**Authors:** Hanning Wang, Juntan Li, Xu Li

**Affiliations:** 1 Department of Orthopedics, The First Hospital of China Medical University, Shenyang, Liaoning Province 110001, P.R. China

**Keywords:** osteosarcoma, prognosis, immune microenvironment, single cell sequencing, nomogram

## Abstract

Background: Osteosarcoma is the most common bone malignancy in teenagers, and warrants effective measures for diagnosis and prognosis. Oxidative stress (OS) is the key driver of several cancers and other diseases.

Methods: The TARGET-osteosarcoma database was employed as the training cohort and GSE21257 and GSE39055 was applied for external validation. The patients were classified into the high- and low-risk groups based on the median risk score of each sample. ESTIMATE and CIBERSORT were applied for the evaluation of tumor microenvironment immune infiltration. GSE162454 of single-cell sequencing was employed for analyzing OS-related genes.

Results: Based on the gene expression and clinical data of 86 osteosarcoma patients in the TARGET database, we identified eight OS-related genes, including MAP3K5, G6PD, HMOX1, ATF4, ACADVL, MAPK1, MAPK10, and INS. In both the training and validation sets, the overall survival of patients in the high-risk group was significantly worse than that in the low-risk group. The ESTIMATE algorithm revealed that patients in the high-risk group had higher tumor purity but lower immune score and stromal score. In addition, the CIBERSORT algorithm showed that the M0 and M2 macrophages were the predominant infiltrating cells in osteosarcoma. Based on the expression analysis of immune checkpoint, CD274(PDL1), CXCL12, BTN3A1, LAG3, and IL10 were identified as potential immune therapy targets. Analysis of the single cell sequencing data also revealed the expression patterns of OS-related genes in different cell types.

Conclusions: An OS-related prognostic model can accurately provide the prognosis of osteosarcoma patients, and may help identify suitable candidates for immunotherapy.

## INTRODUCTION

Osteosarcoma is the most prevalent bone malignancy among adolescents, with a high incidence rate of 8–11/1,000,000/year in the 15–19 years age group [1, 2]. It primarily affects the long bones, including the femur, tibia, and humerus [3]. The second peak of osteosarcoma occurrence is after the age of 50 years [4]. Most patients with osteosarcoma have lung metastases when first diagnosed, and their 5-year survival rate is less than 20% [5]. However, the 5-year survival rate of osteosarcoma patients without lung metastases is almost 70% [6]. Despite treatment options such as surgery and chemotherapy, the 5-year survival rates are overall dismal because of the genetic complexity and instability of these tumors [7, 8]. High-throughput technologies have been used in recent oxidative stress (OS) studies, which have revealed differentially expressed genes (DEGs) such as COL1A2 and matrix metalloproteases (MMPs) between osteosarcoma and normal samples [9, 10]. These sequence data provide a method for early diagnosis and treatment, as well as enable accurate prognosis to improve the outcomes of osteosarcoma patients.

OS is characterized by excessive production of reactive oxygen species (ROS) that cannot be effectively quenched by the cellular antioxidative mechanisms [11, 12]. OS is the underlying pathological basis of degenerative diseases, such as Alzheimer’s disease [13], diabetes [14], and arthritis [15], and cancers, including bladder cancer [16] and breast cancer [17]. Cancer cells are characterized by increased aerobic glycolysis and high levels of ROS [18]. While the high metabolic rates promote cellular migration, survival, and proliferation, ROS-induced oxidative DNA damage initiates oncogenic transformation and subsequent tumor progression. Given the metabolic and signaling aberrations caused by increased ROS levels, the pathways involved in ROS production are promising therapeutic targets for cancer [19]. An increase in oxidative stress and decrease in antioxidant status is observed in primary bone sarcomas [20]. Several studies have also revealed the anti-cancer potential of differential ROS production pathways for treating osteosarcoma *in vitro* and *in vivo* [21–23].

The human immune system has evolved to eradicate pathogens and tumor cells [24], mainly through effector cells such as basophils, macrophages, neutrophils, eosinophils, and natural killer (NK) cells [25]. In contrast, the humoral immune system is less involved in anti-tumor responses, and may even promote tumor growth under certain conditions. The tumor microenvironment (TME) is a complex network of tumor cells, stromal cells, and immune cells. The non-tumor cell populations also have diverse and complex regulatory effects on tumor progression. For instance, an abundance of cancer-associated fibroblasts (CAFs) in the TME is conducive to tumor growth [26], whereas high levels of tumor-associated macrophages (TAMs) are associated with lower metastasis and improved survival in osteosarcoma [27]. Therefore, the role of TME in osteosarcoma prognosis warrants further investigation.

**Figure 1:**
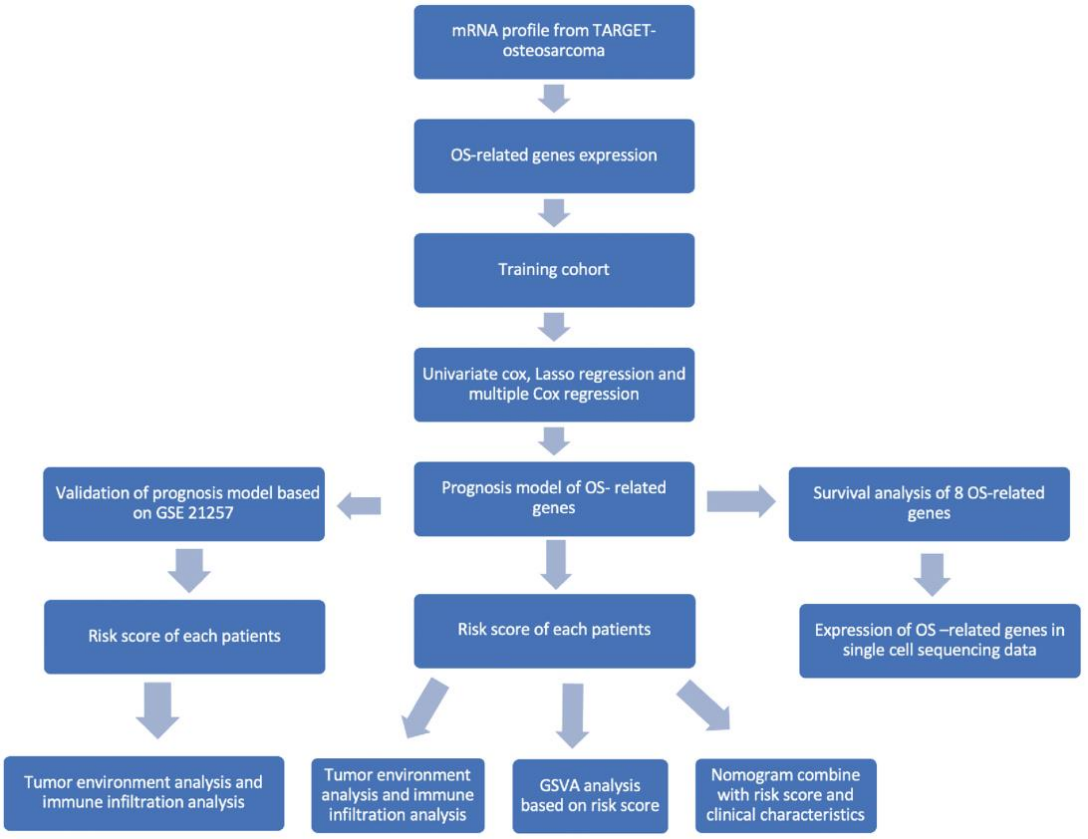
Flowchart of this study.

To this end, we retrieved the clinical and transcriptomic data of osteosarcoma patients from the Therapeutically Applicable Research to Generate Effective Treatments (TARGET) database. The OS-related genes were screened using univariate Cox regression, and a prognostic model was constructed following LASSO and multivariable Cox regression analyses. The accuracy and generalizability of the risk model was validated in an external cohort. The correlation between the risk score and TME was evaluated, and a predictive nomogram was constructed by combining the clinical characteristics and risk score. The flow chart of the study is shown in Figure 1.

## MATERIALS AND METHODS

### Data sources

The mRNA sequencing data and clinical information 86 osteosarcoma patients were retrieved from the TARGET database (https://ocg.cancer.gov/programs/target) to construct an OS-related risk model. A total of 817 OS-related genes were obtained from GeneCards database (https://www.genecards.org/). In addition, the GSE21257 and GSE39055 datasets including 90 osteosarcoma samples was downloaded from the GEO database (https://www.ncbi.nlm.nih.gov/geo/) to validate the risk model. Normalization of fragments per kilobase of exon model per million mapped fragments (FPKM) was performed in the TARGET-OS dataset and Limma package was used to normalize gene expression [28]. The R package sva was used to eliminate batch effect of different databases [29]. The clinical data of patients in the TARGET-OS and validation cohorts are summarized in Table 1. The GSE162454 dataset containing the single-cell sequencing data of six osteosarcoma samples was also retrieved from the GEO database [30].

**Table 1:** Clinical information of training and validation cohorts.

	**TARGET-OS**	**GSE21257**	**GSE39055**
Samples	86	52	38
Age	14.5	16.7	13.5
Female percent	43.0%	36.5%	44.8%
Metastasis percent	25.6%	64.1%	Na
Survival time	4.11	5.71	4.41

### Construction of prognostic model based on OS-related genes

The potential OS-related genes were identified in the TARGET dataset (training cohort) through univariate Cox regression analysis. The GLMNET package [31] from R was then used to perform the least absolute shrinkage and selection operator (LASSO) analysis to remove genes that may lead to overfitting [31, 32]. The seed number for Lasso regression is set to 666, and the penalty coefficient is 0.0868. Finally, the prognostically significant OS-related genes were screened by multivariable Cox regression analysis. The coefficients, hazard ratios (HR), and *p* values of different genes are listed in Table 2. The risk score of each sample from TARGET-OS was calculated using the Predict algorithm, and the patients were divided into high-risk and low-risk groups on the basis of the median risk score. The Kaplan–Meier method was used to evaluate the difference in the survival status of both risk groups, and receiver operating characteristic (ROC) curves for 1-, 3-, and 5-year survival were plotted using the survival ROC package in R to identify the diagnostic value of the risk model [33].

**Table 2:** Eight genes obtained after multivariable Cox regression analysis.

**Gene**	**Coefficient**	**HR**	**HR.95L**	**HR.95H**	***P* value**
MAP3K5	−1.817	0.162	0.057	0.465	0.0007
G6PD	−0.662	0.516	0.228	1.167	0.1120
HMOX1	−0.523	0.593	0.408	0.861	0.0061
ATF4	0.8396	2.315	1.130	4.746	0.0218
ACADVL	1.4825	4.404	2.073	9.357	0.0001
MAPK1	−1.135	0.321	0.138	0.746	0.0083
INS	32.215	9.79E+13	1295.499	7.41E+24	0.0117
MAPK10	−1.540	0.214	0.057	0.795	0.0213

### Validation of OS-related risk model

The performance of the OS-related model was validated in GSE21257 and GSE39055. The risk score for the validation cohort was calculated using the same algorithm as that used for the training cohort, and the samples were divided into the high- and low-risk groups on the basis of the median score for the training cohort. The efficacy of the risk model was evaluated by the Kaplan–Meier method and ROC curve analysis as described.

### Gene set enrichment analysis

The DEGs between the high- and low-risk groups in the TARGET cohort were screened using the Limma package. The significantly enriched gene ontology pathways, namely biological processes (BPs), cellular components (CCs), and molecular functions (MFs), associated with DEGs were identified by gene set variation analysis (GSVA) packages in R software with |log2FC|>1 and adj. *P* value < 0.05 as thresholds [34].

### Immune analysis based on OS-related genes

The Estimation of Stromal and Immune cells in Malignant Tumor tissues (ESTIMATE) package in R software was used to calculate tumor purity, stromal score, and immune score in the two risk groups [35]. The CIBERSORT algorithm was used to calculate the proportion of 22 infiltrating immune cell populations in the training and verification cohorts [36] and in the high- and low-risk groups. Some potential immune checkpoints such as PD1 (PDCD1), PDL1 (CD274), IL10, CTL4, BTN3A1, CXCL12, and LAG3, and calculated the CYT score (GZMA and PRF1) were displayed using boxplots to illustrate their expression across high- and low-risk groups, revealing potential immune therapeutic targets. The gene mutation data was obtained from the TCGA database, and the maftools package was used to visualize the mutation data.

### Construction of a predictive nomogram based on the risk model

The prognostic model was integrated with the clinical characteristics including gender, race, metastasis, and risk score to build a nomogram using the Survminer package in R. The predictive accuracy of the nomogram was validated by C-index and comparison of the fitting degree between the observed and optimized values was performed.

### OS-related gene expression in single-cell sequencing database

The expression of OS-related genes at the single cell level was analyzed in the GSE162454 dataset. Six osteosarcoma samples were analyzed using the Seurat package (Version 4.0) [37]. Poor quality cells (genes >8000, genes <500, or >10% genes mapping to mitochondrial genome) were excluded. The expression data were transformed to the log scale and normalized for scaling the sequencing depth. T-Stochastic neighbor Embedding (T-SNE) plots were used to visualize cell clusters.

### Immunohistochemistry and hematoxylin-eosin staining

OS-related genes were experimentally verified by immunohistochemistry (IHC). Three osteosarcoma tissues and peri-tumor tissues were collected from patients at First Affiliated Hospital of China Medical University. A consent form was signed by each participant. The present study was approved by the ethics committee of the hospital with the registration number 2019-285-2.

Hematoxylin-eosin (HE) staining was performed to identify histology type of tissues. Tissues were fixed and paraffinized before IHC staining. Antigens were retrieved using citrate antigen retrieval buffer. Endogenous peroxidase was blocked by 3% hydrogen peroxide solution, incubated at room temperature for 25 min in the dark. In the histochemical area, 3% BSA (Bovine Serum Albumin, Servicebio) was added dropwise to cover the tissue evenly, and it was sealed at room temperature for 30 min. Sections were incubated overnight at 4°C in a humidified chamber with corresponding antibodies. Antibody applications were as follows: ACADVL (Proteintech, 1:200), ATF4 (Servicebio, 1:2000), HMOX1 (Servicebio, 1:500), MAPK10 (Servicebio, 1:200), INS (Servicebio, 1:200). Secondary antibody (HRP-labeled) corresponding to the primary antibody was added to cover the tissue area and slides were incubated at room temperature for 50 minutes. Freshly prepared DAB (3,3′-Diaminobenzidine) chromogenic were used for color development in the IHC area. Nuclei were counterstained with hematoxylin for about 3 min. Finally, microscopic examination and image acquisition analysis were performed.

### Statistical analysis

Statistical analyses were performed using the R software (Version 4.1.3). Two-tailed Student’s *t*-test and Wilcox test were performed to compare gene expression or scores between two independent groups. Kaplan–Meier survival analysis, LASSO regression analysis, and multivariable Cox regression analysis were performed to identify prognostic genes. Forest plots of nomogram were visualized using forest algorithm. *P* value < 0.05 was considered statistically significant.

### Availability of data and materials

The source data and R code of the manuscript was uploaded in OSF (https://doi.org/10.17605/OSF.IO/238HP).

## RESULTS

### Identification of OS-related genes in osteosarcoma

A total of 817 OS-related genes were identified from the GeneCards database, and their expression matrix was extracted from the TARGET database. Thereafter, 89 OS-related genes were identified by univariate cox regression analysis, using *p* < 0.05 as the cut off (Supplementary Table 1); of these, 12 genes were screened on the basis of their predictive value using LASSO regression (Supplementary Figure 1 and Supplementary Table 2).

### Construction of an OS-related prognostic model

**Figure 2:**
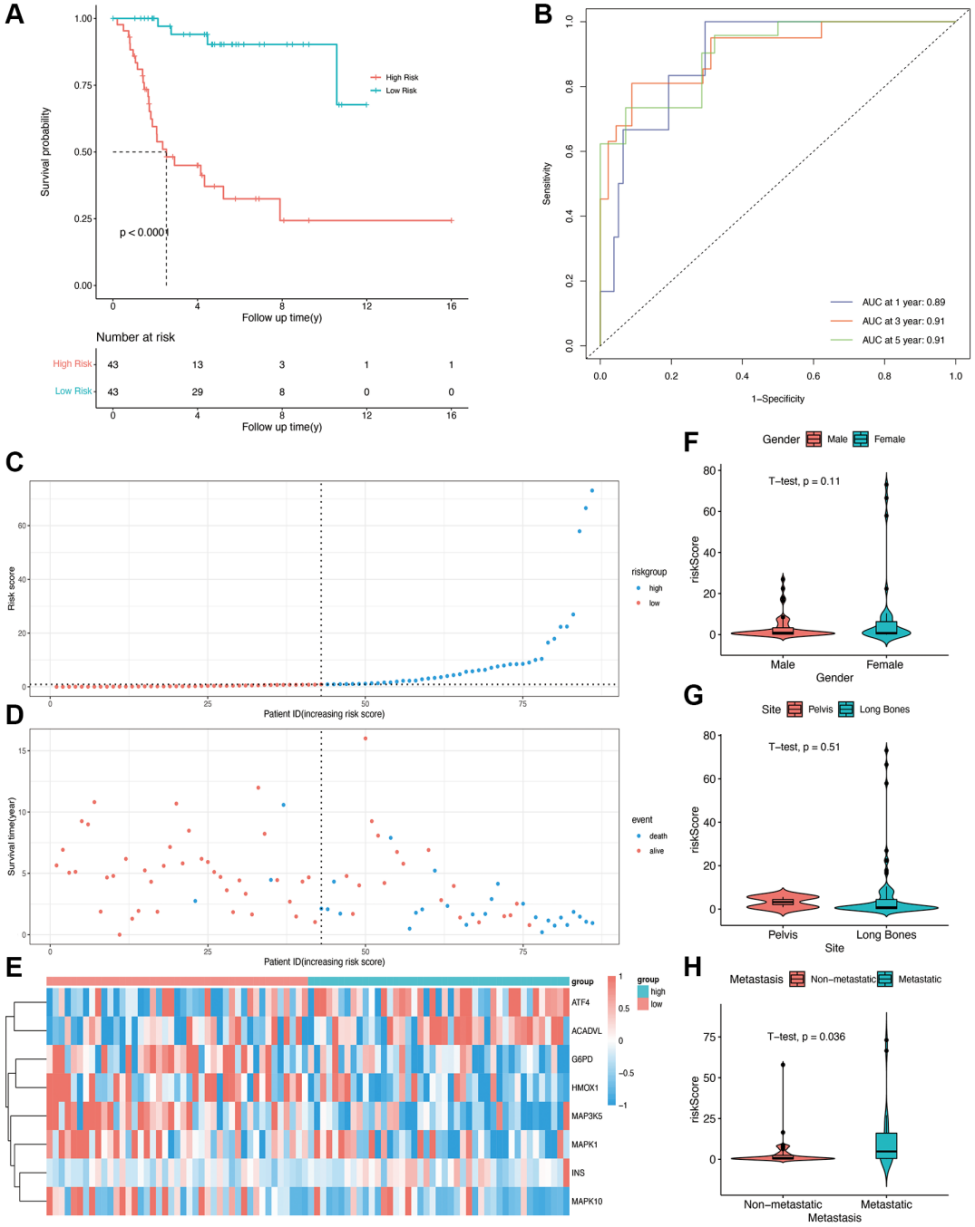
**Construction of OS-related prognostic model for osteosarcoma in the TARGET cohort.** (**A**) Kaplan-Meier curves showing the overall survival in the high-risk and low-risk groups (*p* < 0.0001). (**B**) AUCs for 1-, 3-, and 5-year survival according to the ROC curves. (**C**) The risk score curve of each patient. (**D**) The distribution of survival status. (**E**) The heatmap of eight OS-related genes between the high- and low-risk groups. (**F**, **G**, **H**) Risk scores in the gender, site, and metastasis subgroups.

A multivariable Cox regression model was constructed, and eight prognostic OS-related genes were obtained using the stepwise regression function (Table 2). The risk score of each patient was calculated, and the patients were stratified into high-risk and low-risk groups depending on the median score. The patients in the low-risk group showed better overall survival compared with those in the high-risk group (Figure 2A, *p* < 0001). The time-dependent ROC curve was then plotted to evaluate the predictive efficiency of the prognostic model. The areas under curve (AUCs) for 1-, 3-, and 5-year survival were 0.89, 0.91, and 0.91, respectively (Figure 2B), suggesting that the model can accurately predict the overall survival of osteosarcoma patients. The risk score distribution of patients in the high- and low-risk groups is shown in Figure 2C and 2D, and the expression levels of OS-related genes in each patient are shown in the form of a heatmap in Figure 2E. We calculated the risk score in different subgroups, and did not observe any significant effect of gender or the site of osteosarcoma on risk scores (Figure 2F and 2G). However, patients with metastasis had a higher risk score than those without metastasis (*p* = 0.036, Figure 2H).

### Validation of prognostic model

**Figure 3:**
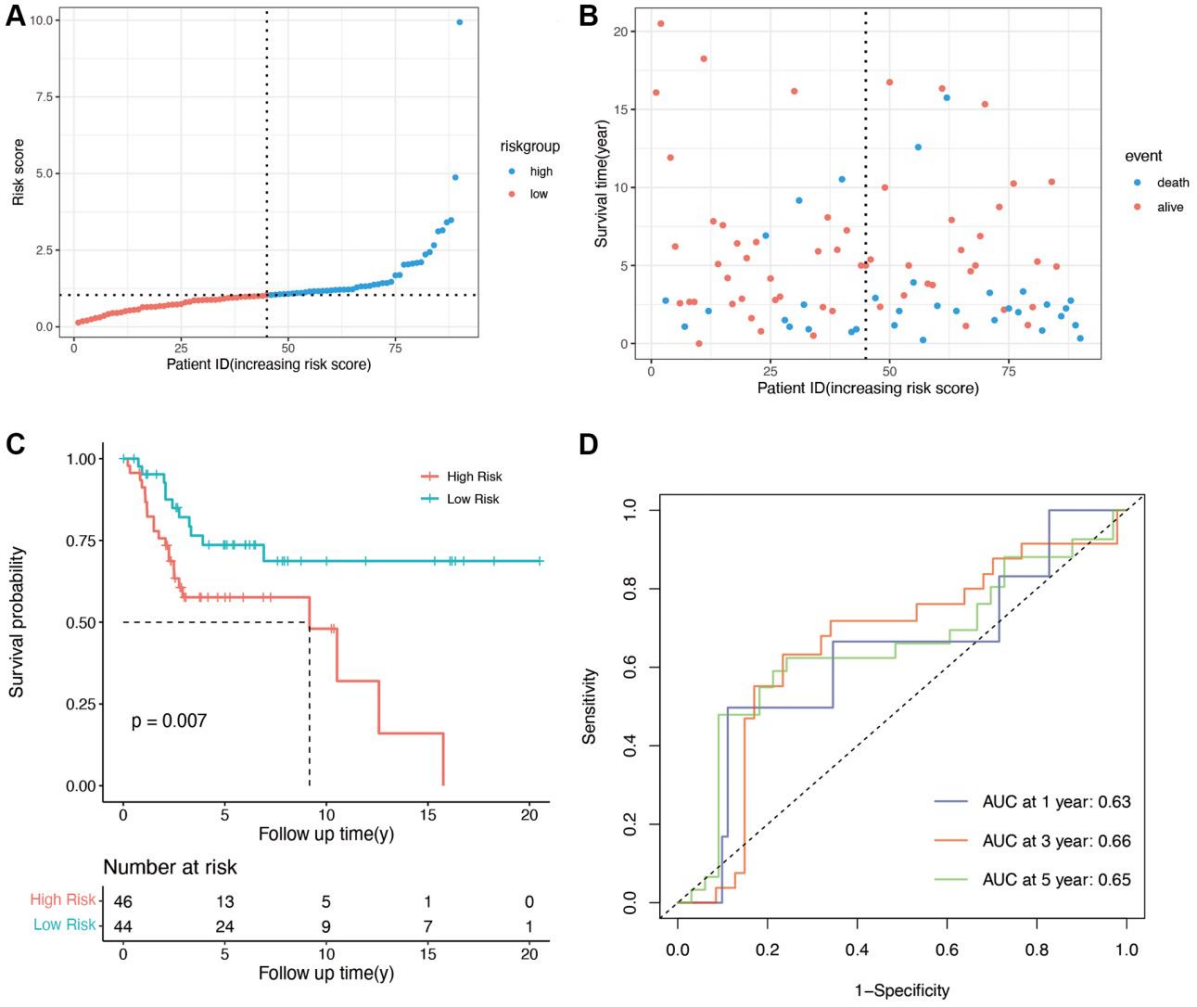
**Validation of OS-related genes in the GSE21257 and GSE39055 cohort.** (**A**) Validation group was divided into high- and low-risk groups using the median risk score as the cutoff value. (**B**) the relationship between risk score and survival time and status of patients. (**C**) K-M plot of high and low risk groups in validation group (*p* = 0.007). (**D**) AUCs for 1-, 3-, and 5-year survival according to ROC curves.

To validate the predictive ability of the OS-related prognostic model, the independent dataset GSE21257 and GSE39055 were used for the validation of our risk model. The patients in the validation cohort were also grouped into the high- and low-risk groups based on the median score in the training cohort. As shown in Figure 3A, and 3B, compared with low-risk group, the high-risk group had lower survival time. The K-M plot of validation group was shown in Figure 3, there was significant difference in high and low risk group (*p* = 0.007). The areas under the curve for prediction of 1-, 3-, and 5-years survival of patients were 0.63, 0.66 and 0.65, respectively (Figure 3D).

### Survival analysis and functional enrichment of the prognostic OS genes

**Figure 4:**
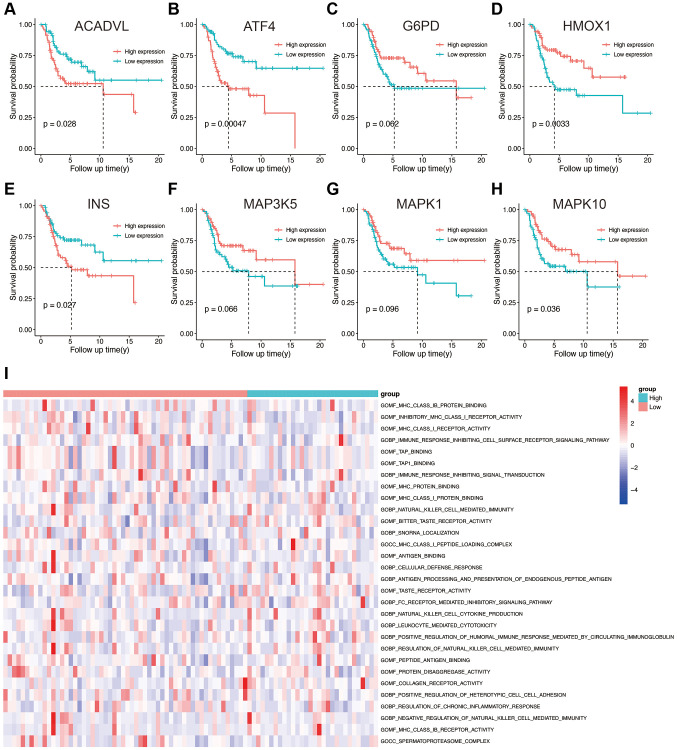
(**A**–**H**) Kaplan-Meier survival analysis of eight OS-related genes. (**I**) Results of GSVA in the high- and low-risk groups. Abbreviations: GO: gene ontology; BP: biological process; CC: cellular components; MF: molecular functions.

Based on the expression level of each gene in the prognostic model, the patients were divided into high- and low-expression groups. The survival curves corresponding to the genes are shown in Figure 4A–4H. Low expression levels of ACADVL (*p* = 0.028), ATF4 (*p* = 0.00047), and INS (*p* = 0.027) were associated with improved survival, whereas high expression of HMOX1 (*p* = 0.0033) and MAPK10 (*p* = 0.036) showed better prognosis. To further explore the underlying molecular mechanisms of the prognostic model, we identified 106 differentially enriched GO terms between the high- and low-risk groups in the TARGET database with |log2FC|>1 and adj. *p* value < 0.05 as the cut-offs. The top 50 enriched GO terms obtained from GSVA are shown in Figure 4I, and these included many immune-related terms such as immune response, positive and negative regulation of T cells, and leukocyte-mediated cytotoxicity. This suggested that the OS-related prognostic genes have an impact on TME and immune infiltration in osteosarcoma.

TARGET database with |log2FC|>1 and adj.*p* value < 0.05 as the cut-offs. The top 50 enriched GO terms obtained from GSVA are shown in Figure 4I, and these included many immune-related terms such as immune response, positive and negative regulation of T cells, and leukocyte-mediated cytotoxicity. This suggested that the OS-related prognostic genes have an impact on TME and immune infiltration in osteosarcoma.

### Validation of survival signatures of OS-related genes

**Figure 5:**
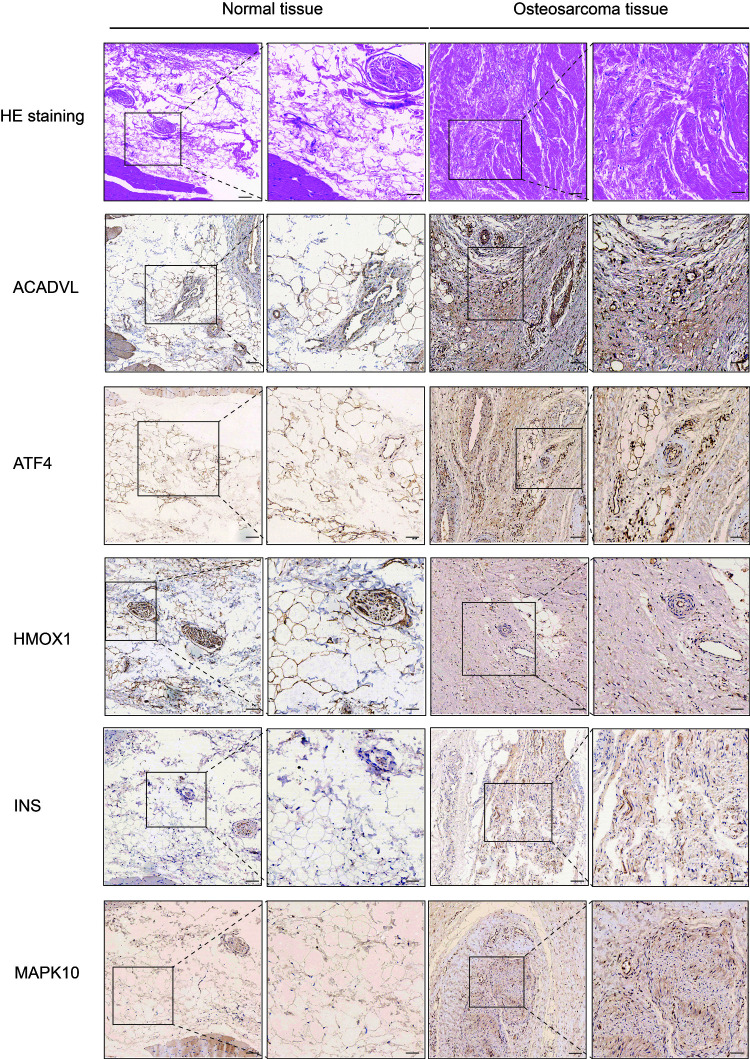
**Hematoxylin eosin (HE) staining and immunohistochemistry (IHC) staining of normal and osteosarcoma tissue.** Scale bar, 100 μm (left panel) and 50 μm (right panel).

We performed IHC among OS-related genes with significantly difference using corresponding antibodies. The IHC results illustrated that the expression of AVADVL, ATF4, INS and MAPK10 was higher in osteosarcoma tissues compared to normal tissues (Figure 5). However, the expression of HMOX1 was lower in osteosarcoma tissues, which was in line with the prediction of Kaplan-Meier survival curve.

### Estimation of tumor immune microenvironment

**Figure 6:**
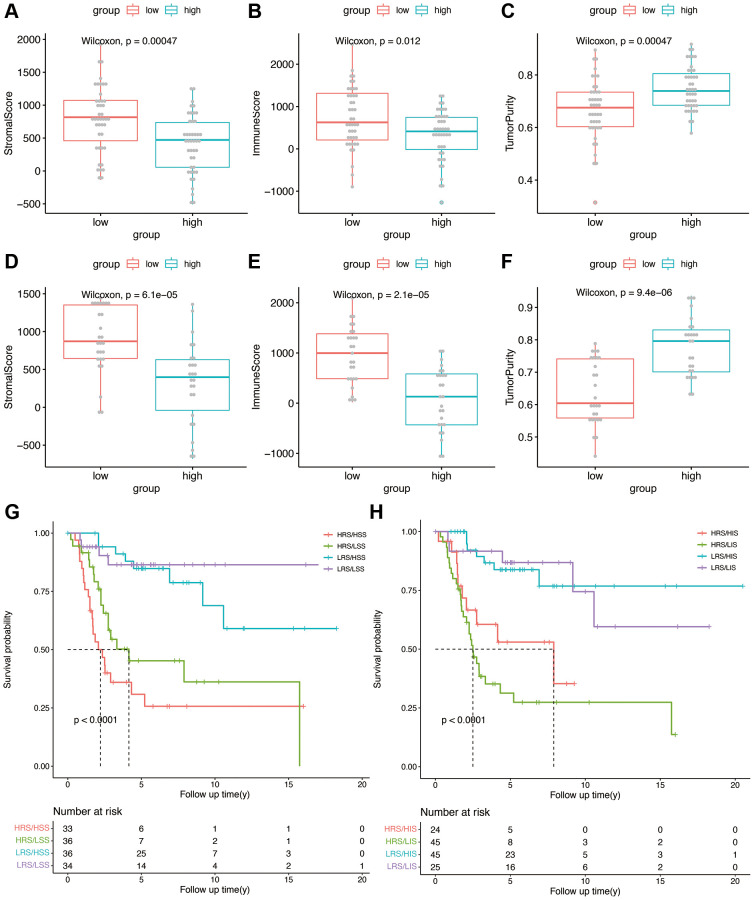
(**A**–**C**) Stromal score, immune score, and tumor purity in the high- and low-risk groups in the TARGET cohort. (**D**–**F**) Stromal score, immune score, and tumor purity in the high- and low-risk groups in the validation cohort. (**G**) Kaplan-Meier analysis combining risk score and stromal score. (**H**) Kaplan-Meier analysis combining risk score and immune score.

We calculated the stromal score, immune score, and tumor purity in each sample in the TARGET and GSE21257 datasets using the ESTIMATE algorithm. We also compared the abovementioned parameters between the high- and low-risk groups, and found that patients in the high-risk group with lower stromal score had better overall survival, compared with those with higher stromal scores (*p* < 0.0001, Figure 6A, 6D). However, a higher immune score was associated with better overall survival as opposed to a lower immune score in the high-risk group (*p* < 0.0001, Figure 6B, 6E). High risk group of training and validation group had higher tumor purity than low risk group (Figure 6C, 6F). Thus, OS-related prognostic genes are associated with the immune microenvironment of osteosarcoma, and may be able to predict the efficacy of immunotherapy. Then risk score was combined with stromal score and immune score as shown in Figure 6G and 6H, which illustrated the survival relationship of tumor immune microenvironment score.

### Tumor-infiltrating immune cells using OS-related risk score

**Figure 7:**
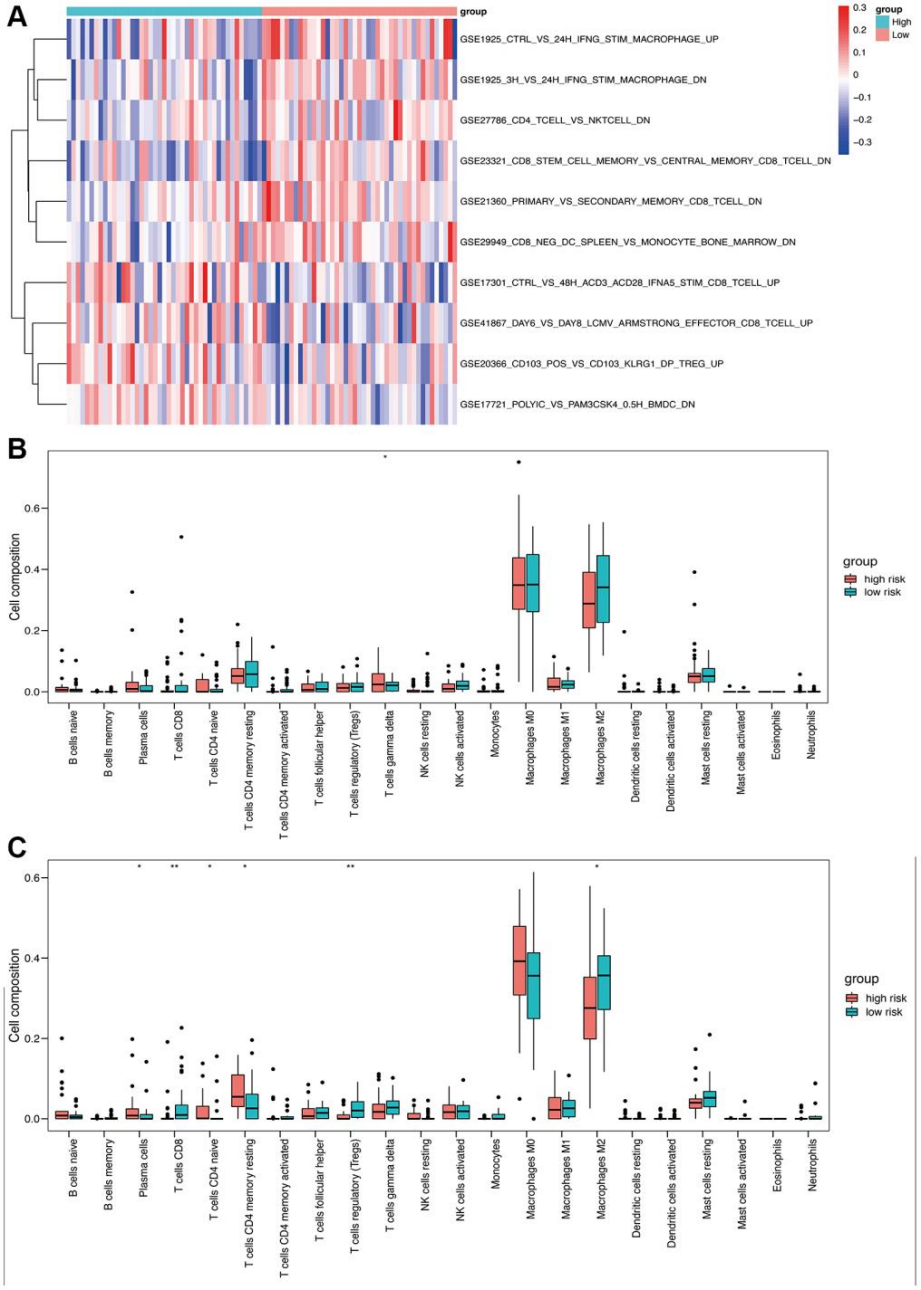
**Immune analysis based on high- and low-risk groups.** (**A**) Immune-related pathway GSVA between high- and low-risk groups. (**B**) Box plot of 22 immune cells in the high- and low-risk groups of the training cohort. (**C**) Box plot of 22 immune cells in the high- and low-risk groups of the validation cohort.

Immune-related pathway GSVA revealed that macrophages were well enriched between high- and low-risk groups (Figure 7A). The CIBERSORT algorithm was used to evaluate the infiltration of 22 immune cell populations in the TARGET and GSE21257 osteosarcoma samples (Figure 7B and 7C). M0 and M2 macrophages were the top 2 infiltrating cell types in both cohorts. In the TARGET cohort, there was a significant difference in the abundance of infiltrating gamma delta T cells between the high- and low-risk groups (Figure 7B). In contrast, significant differences were observed in the infiltration of plasma cells, CD8 T cells, CD4 naïve T cells, CD4 memory T cells, regulatory T cells, and M2 macrophages between the high- and low-risk groups in the GSE21257 cohort (Figure 7C). These results suggested that the OS-related model is associated with the immunological status of the osteosarcoma microenvironment.

### Sensitivity to immunotherapy and tumor mutation burden analysis

**Figure 8:**
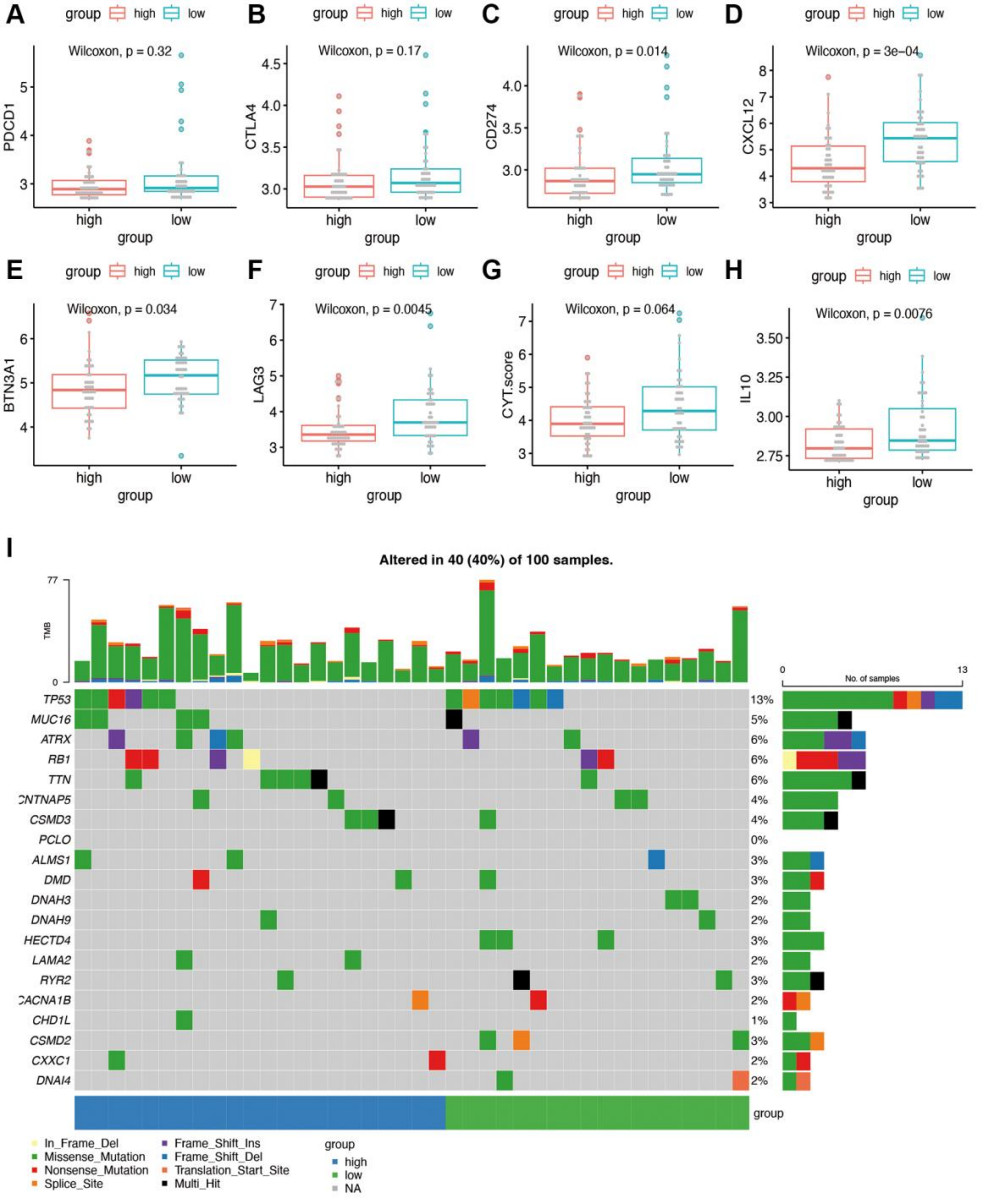
**Immunotherapy sensitivity and tumor mutation burden analysis.** (**A**–**H**) Expression levels of representative immune checkpoints between high and low risk group. (**I**) Tumor mutation analysis of high and low risk group.

To explore sensitivity to immunotherapy, we performed further expression analysis for the possible immunotherapy target. The expression of several representative immune checkpoint genes is shown in Figure 8A–8H. We found that the expression of CD274 (PDL1), CXCL12, BTN3A1, LAG3, and IL10 was significantly decreased in the high-risk group of our model. No significant difference was found in the term of PDCD1(PD1), CTLA4 and CYT score. Figure 8I showed the gene mutation profiles of the high- and low-risk groups. With the application of maftools package, it can be observed that the proportion of mutated genes is not high, with p53 being the most frequently mutated gene. There is no significant difference between the high- and low-risk groups in terms of gene mutations.

### Construction and validation of the nomogram

**Figure 9:**
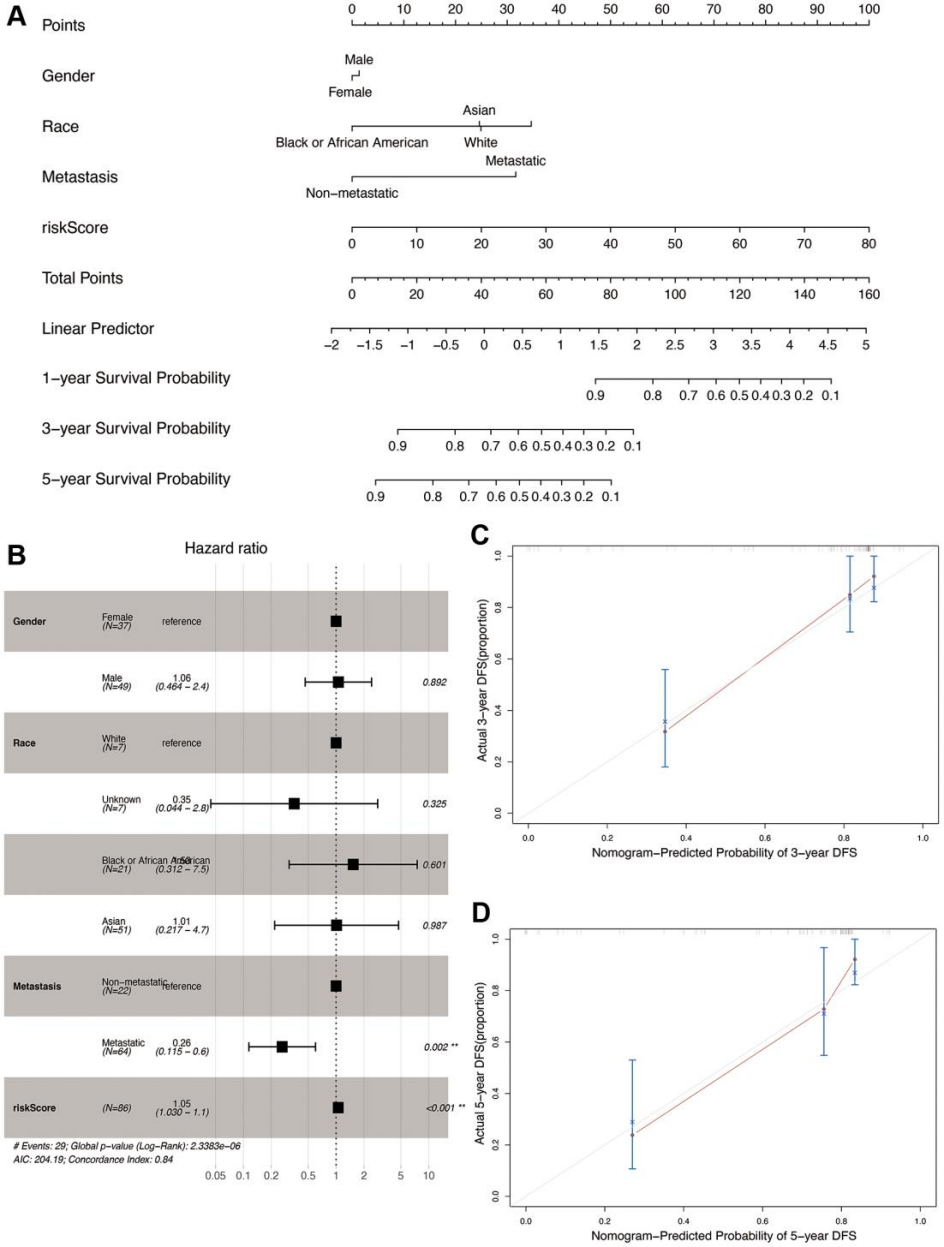
**Construction and validation of the nomogram in the TARGET cohort.** (**A**) Nomogram combining OS-related risk score and clinical characteristics for predicting 1-, 3-, and 5-year overall survival of osteosarcoma patients. (**B**) Forest plot showing results of multivariable cox regression of the nomogram. (**C**, **D**) The calibration curves for 3- and 5-year overall survival probability.

We constructed a nomogram by integrating the OS-related prognostic model and clinical characteristics including gender, race, and metastasis status. As shown in Figure 9A, each item was assigned a score and the total score of each patient predicted the probability of survival in 1, 3, and 5-years. The Forest plot of the nomogram revealed that metastasis and risk score showed significant prognostic value in the cohort (Figure 9B). The accuracy of the nomogram was validated in the TARGET cohort (Figure 9C and 9D), and the results indicated a good fit between the predicted and actual 3- and 5-year survival rates.

### Single cell expression of OS-related mRNAs

**Figure 10:**
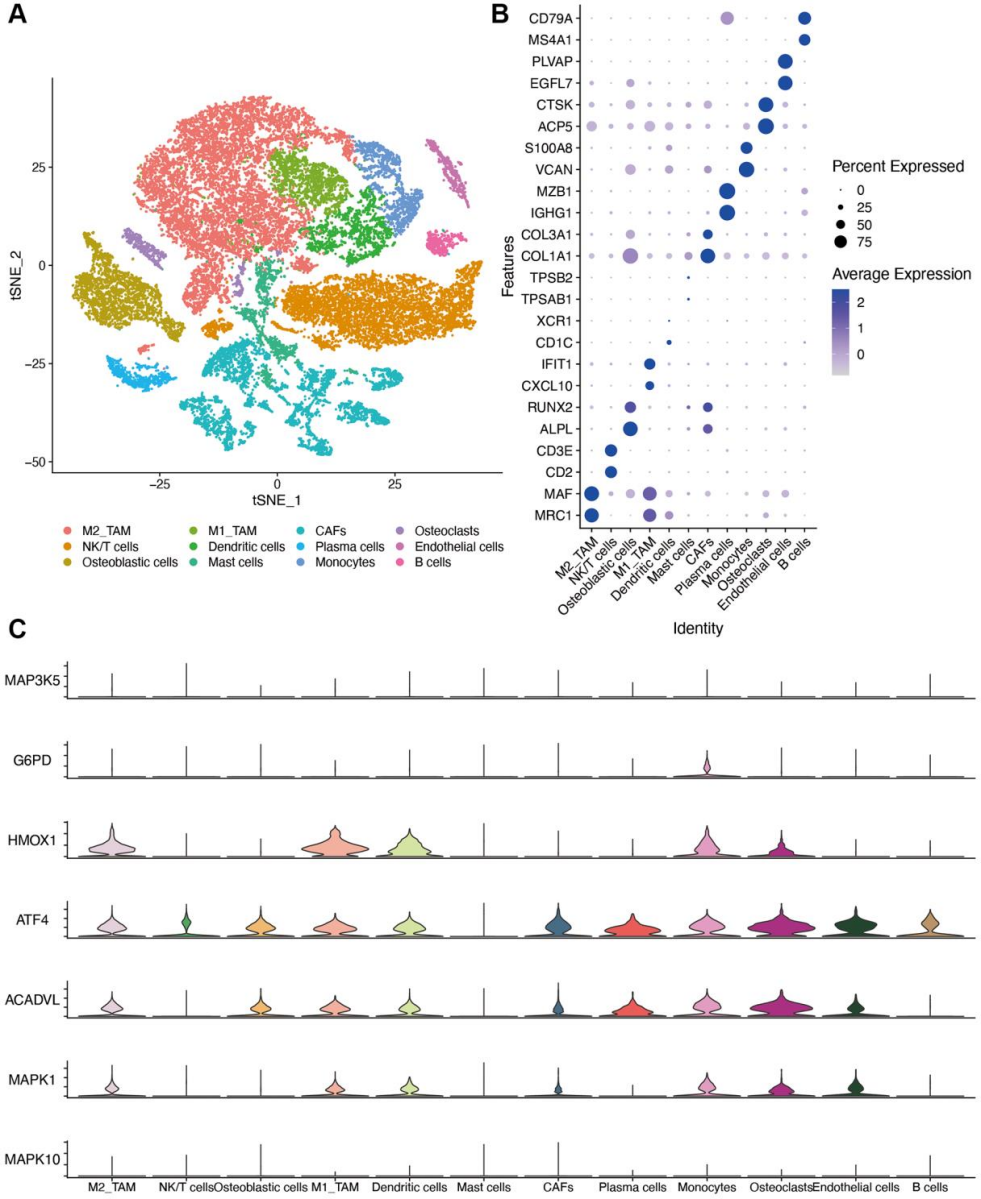
**Single-cell sequencing dataset analysis.** (**A**) The TSNE plot of 12 cell clusters of GSE162454. (**B**) Dot plot showing the expression levels of marker gene in single cell sequencing data. (**C**) violin plot of OS-related genes in different cell types. Abbreviations: CAF: cancer associated fibroblasts; TAM: tumor associated macrophages.

We identified 23 immune cell subpopulations in the GSE162454 dataset by TSNE analysis at first. Based on the specific markers, we identified NK/T cells, CAFs (cancer associated fibroblasts), endothelial cells, plasma cells, osteoblastic cells, osteoclasts, B cells, M2 TAMs (tumor associated macrophages), M1 TAMs, monocytes and mast cells (Figure 10A). The expression of marker genes for different cell clusters was displayed in dot plot format in Figure 10B. The expression of OS-related genes was compared in different cell types. Because of the dropout sign of single-cell sequencing, INS was not detected in the GSE162454 dataset. As shown in Figure 10C, HMOX1 were expressed at higher levels in TAMs and osteoclasts. ATF4 and ACADVL are highly expressed in myeloid cells as well as other cell types, which was suggestive of their roles in osteosarcoma progression.

## DISCUSSION

Nearly 10 million adolescents are diagnosed with osteosarcoma each year [38]. Because of the anatomical features of bone tissues, chemotherapeutics have not achieved satisfactory response in bone malignancies [39]. OS, which is reflected in the excessive accumulation of ROS, frequently initiates tumorigenesis by promoting lipid oxidation and DNA damage [40]. Therefore, it is crucial to identify the OS-related genes in osteosarcoma and other cancers to increase the repertoire of therapeutic targets. In the present study, we analyzed the high-throughput RNA-sequencing data and clinical information of 86 osteosarcoma patients, and identified eight prognosis-related genes, including MAP3K5, G6PD, HMOX1, ATF4, ACADVL, MAPK1, MAPK10, and INS. An OS-related risk model was developed for predicting patient prognosis. With the combination of expressions of these eight genes, the risk scores had better prognostic performance than with individual gene expressions. Because the OS-related prognostic genes were enriched in immune-related functions, we analyzed the relationship between the risk model and the immunological characteristics of the TME, and observed significant differences in the infiltrating immune cell populations between the high- and low-risk groups. Furthermore, analysis of single cell sequencing results indicated that the OS-related genes were expressed in different cell types, which offers insights into potential treatment strategies for osteosarcoma.

In the field of osteosarcoma, multiple gene sets related to patient prognosis, such as ferroptosis-related genes, immune-related genes, and lipid metabolism genes, have been found [41–43]. However, whether OS-related genes can predict patient outcomes has not been investigated. In this study, the risk scores of patients were calculated based on the expressions of eight OS-related genes, and patients were divided into high- and low-risk groups according to the median number. The difference in survival time between the two groups was statistically significant.

MAP3K5 is a serine/threonine kinase that mediates the MAP kinase signal transduction pathway [44] in different cancer types, such as pancreatic cancer [45], prostate cancer [46], and thyroid cancer [47]. It is also a ferroptosis-related gene associated with increased ROS accumulation [48]. G6PD is the first and the rate-limiting enzyme in the pentose phosphate pathway, as well as an antioxidant enzyme involved in the ribose 5-phosphate pathway for nucleotide synthesis. Wang et al. found that G6PD inhibition upregulated ROS levels in osteosarcoma cells and induced endoplasmic reticulum (ER) stress, which is consistent with the decreased survival rates observed in the G6PD^low^ group in our cohort. HMOX1 is a cytoprotective enzyme and its overexpression in U-2OS cells has been reported to significantly decrease cell proliferation rates by inducing ferroptosis [49]. Further, ATF4 is a master transcriptional regulator of amino acid metabolism and stress responses [50], and induces apoptosis in response to persistent stress conditions through post-transcriptional modifications. ACADVL is an inner mitochondrial membrane protein and catalyzes the first step of the mitochondrial fatty acid beta-oxidation pathway [51]. However, there are currently no reports on the possible role of ACADVL in osteosarcoma.

The mitogen-activated protein kinase (MAPK) family of proteins integrate various upstream signals to regulate multiple cellular functions, including proliferation, differentiation, and survival. MAPK1 is overexpressed in breast cancer [52], lung cancer [53], ovarian cancer [54], and other malignancies. Several studies have shown that MAPK1 is targeted by non-coding RNAs to modulate the invasion and proliferation of osteosarcoma cells [55, 56]. MAPK10 is also associated with the prognosis of renal cell carcinoma [57] and hepatocellular carcinoma [58]. INS encodes insulin, a peptide hormone that plays a vital role in the regulation of carbohydrate and lipid metabolism, and is aberrantly expressed in various diseases. Gene expression microarray analysis in a previous study identified INS as one of the core genes in osteosarcoma with pulmonary metastasis [59]. In this study, we found significant differences between the prognosis of patients with low and high expression levels of ACADVL, ATF4, HMOX1, INS, and MAPK10, indicating that these genes are potential therapeutic targets of osteosarcoma.

We further validated the OS-related risk model in an external cohort, and the ROC curve analysis showed high predictive accuracy of the model in both the training and validation cohorts. In addition, the nomogram integrating the risk score and clinical characteristics also reliably predicted the prognosis of osteosarcoma.

The immune microenvironment has been illustrated to have a significant effect on the prognosis of several diseases. Previous studies have shown that lower immune scores are associated with worse prognosis in osteosarcoma [60, 61]. GO analysis based on the risk score in our study illustrated several immune related terms. We found that the high-risk group had significantly higher tumor purity but lower stromal score and immune score in both the training and validation sets, which correlated with a worse prognosis. Myeloid cells, including macrophages, microglia, myeloid-derived suppressor cells, dendritic cells and neutrophils [62], are the predominant infiltrating immune cell populations in the TME, and regulate immune and therapeutic responses [63]. Xiao et al. developed a macrophage-associated risk model for osteosarcoma, which included MAP3K5 as one of the prognosis-related genes [61]. Our results also found that the potential sensitive immune checkpoint such as PDL1, CXCL12, BTN3A1, LAG3, and IL10, were differently expressed between high- and low- risk groups, which provide new therapy target.

Analysis of a single cell sequencing dataset also showed that most of the OS-related genes were highly expressed in myeloid cells in addition to their high expression in osteoblasts and osteoclasts. This warrants further investigation of the function of these genes in osteosarcoma. To summarize, our OS-related risk model is a potential indicator of the prognosis of osteosarcoma patients.

Our study has some limitations that ought to be considered. First, the sample size in our study was small compared with that in other similar studies. Second, the clinical data of the patients were not sufficient, and some key information such as the tumor stage and pathological type could not be included in the nomogram. Third, this study was based on sequencing data and bioinformatics analysis, and the results will have to be validated through *in vitro* and *in vivo* studies.

## CONCLUSIONS

We constructed a prognostic risk model for osteosarcoma based on eight OS-related genes. The model could accurately predict patient prognosis and its performance was validated in an external cohort. In addition, the risk groups classified on the basis of the gene risk score showed significant differences in the immune microenvironment, some immune checkpoints showed difference between high and low risk groups. OS-related genes were highly expressed in myeloid cells. In conclusion, OS-related genes possibly regulate the immune responses in osteosarcoma, and may predict patient response to immunotherapies.

## Supplementary Materials

Supplementary Figure 1

Supplementary Tables
