# Supplementary figures and images for: Construction and validation of an oxidative-stress-related risk model for predicting the prognosis of osteosarcoma
Source: Aging (Albany NY). 2023 Jun 2;15(11):4820–43. doi: 10.18632/aging.204764 (PMC10292890; doi:10.18632/aging.204764)

SUPPLEMENTARY FIGURE

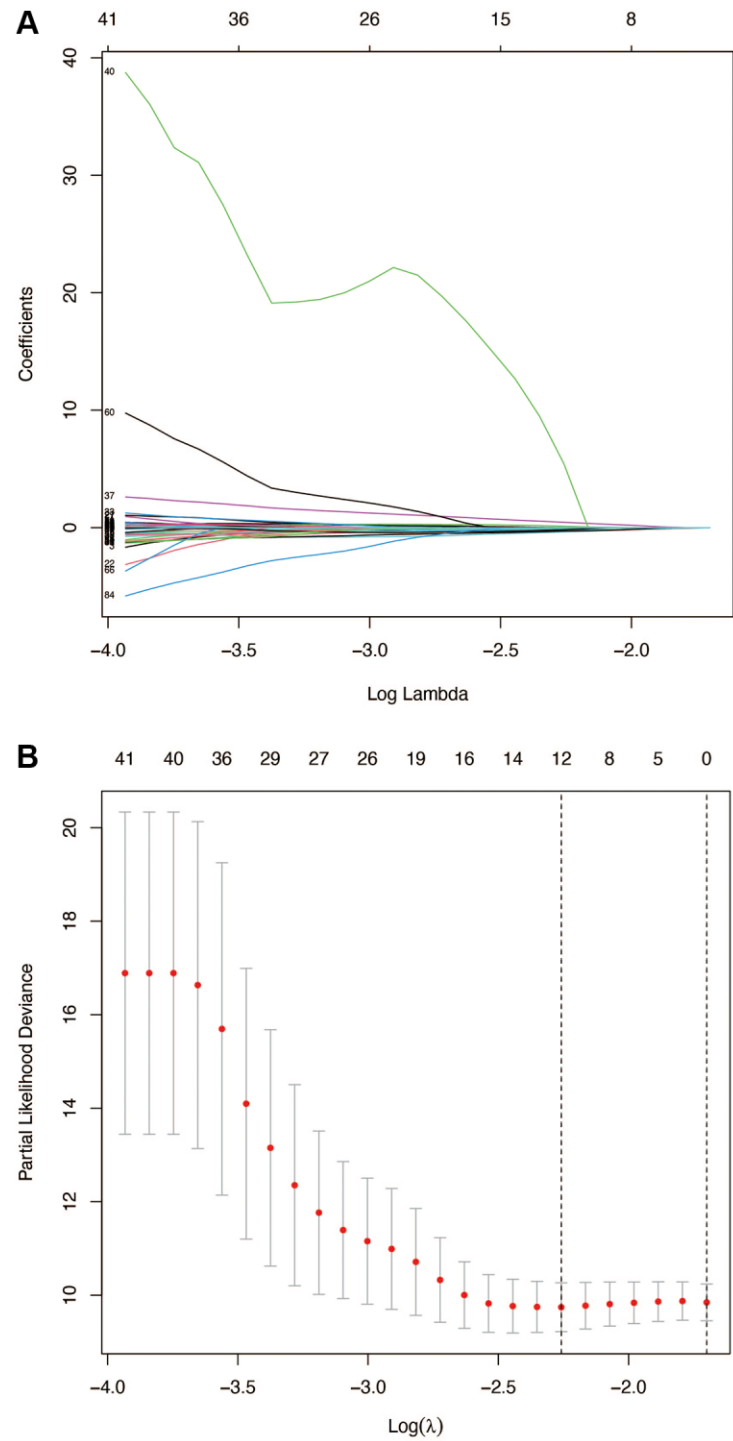

Supplementary Figure 1. LASSO regression analysis with optimal lambda.

Supplement: Supplementary Figure 1 [file aging-15-204764-s001.pdf]
