# Supplementary material for: Construction and validation of an oxidative-stress-related risk model for predicting the prognosis of osteosarcoma
Source: Aging (Albany NY). 2023 Jun 2;15(11):4820–43. doi: 10.18632/aging.204764 (PMC10292890; doi:10.18632/aging.204764)
Supplement: Supplementary Tables [file aging-15-204764-s002.pdf]

SUPPLEMENTARY TABLES

Supplementary Table 1. 89 OS-related genes in osteosarcoma identified by univariate cox regression analysis.

Gene	HR	HR.95L	HR.95H	*p*-value
CAT	0.332	0.164	0.671	0.002
HMOX1	0.721	0.530	0.981	0.037
MAPK1	0.388	0.178	0.848	0.018
G6PD	0.376	0.188	0.752	0.006
TXN2	0.422	0.204	0.871	0.020
GPX1	0.636	0.412	0.980	0.040
ACADVL	2.886	1.374	6.065	0.005
MAP3K5	0.286	0.120	0.682	0.005
CCL2	0.553	0.363	0.842	0.006
INS	15608084289545.4	15654.959	15561349814940800000000	0.004
ATF4	1.845	1.087	3.131	0.023
EIF2AK3	2.416	1.197	4.878	0.014
FMO3	0.388	0.157	0.964	0.041
IL10	0.020	0.001	0.629	0.026
ACE	0.305	0.095	0.981	0.046
PPARG	0.426	0.257	0.708	0.001
NUDT1	1.965	1.146	3.369	0.014
TLR4	0.495	0.261	0.938	0.031
MAOB	1.519	1.015	2.274	0.042
SFXN4	3.580	1.593	8.042	0.002
ATM	1.735	1.054	2.857	0.030
VCAM1	0.589	0.405	0.858	0.006
GPX7	2.302	1.262	4.197	0.007
KCNJ5	0.189	0.061	0.587	0.004
CD36	1.601	1.156	2.217	0.005
CYP2C19	5205.861	6.620	4093638.921	0.012
MB	1.169	1.015	1.347	0.030
BLVRB	0.519	0.312	0.862	0.011
MAP2K4	0.523	0.287	0.953	0.034
EME2	4.008	1.028	15.627	0.046
AGTR1	0.516	0.294	0.903	0.020
CYGB	1.624	1.034	2.549	0.035
MRPS23	0.309	0.114	0.837	0.021
PIK3CG	0.289	0.112	0.748	0.010
ADH5	0.344	0.147	0.807	0.014
CBS	2.188	1.269	3.771	0.005
ANXA5	0.461	0.223	0.952	0.036
TERT	4.444	2.120	9.314	0.000
MAP2K7	2.826	1.346	5.931	0.006
EGFR	0.610	0.401	0.928	0.021
TPD52	2.577	1.509	4.399	0.001
NDUFV1	2.627	1.082	6.378	0.033
TLR2	0.357	0.162	0.786	0.011
CYP11B2	402875.664	13.324	12181370764.833	0.014

PGD	0.364	0.183	0.726	0.004
MAPKAPK3	0.508	0.275	0.940	0.031
INSR	2.307	1.231	4.326	0.009
NDUFB9	1.478	1.053	2.074	0.024
CRAT	0.471	0.278	0.798	0.005
TNFRSF1A	0.360	0.199	0.653	0.001
PTPN1	0.432	0.189	0.984	0.046
LOX	1.745	1.222	2.491	0.002
SUOX	0.294	0.090	0.959	0.042
BMP2	1.363	1.007	1.844	0.045
ALDH1A1	0.619	0.399	0.959	0.032
NOL3	1.999	1.190	3.359	0.009
RPS27A	2.068	1.044	4.096	0.037
AOC3	2.115	1.299	3.443	0.003
CYP2C8	2.816	1.142	6.942	0.025
MYC	2.246	1.439	3.505	0.000
CYP2A6	0.001	0.000	0.706	0.040
PRNP	0.506	0.285	0.898	0.020
PAH	69.244	1.223	3921.661	0.040
CYP27A1	0.584	0.375	0.910	0.018
FAAH	1.696	1.115	2.580	0.014
OGDH	0.288	0.126	0.660	0.003
NDUFB10	0.529	0.290	0.966	0.038
TG	2.133	1.092	4.166	0.027
PTS	2.438	1.203	4.942	0.013
MAPKAP1	0.257	0.085	0.771	0.015
CASP1	0.588	0.358	0.967	0.036
AS3MT	34.423	4.283	276.687	0.001
CHEK1	1.916	1.056	3.477	0.032
ADRB1	2.475	1.362	4.497	0.003
EPHX2	2.183	1.140	4.180	0.019
SCARB1	1.571	1.093	2.258	0.015
PXN	0.390	0.164	0.924	0.032
PRKD2	0.377	0.146	0.975	0.044
KCNMA1	2.430	1.382	4.272	0.002
UGT1A6	4651.907	3.283	6591366.350	0.023
TREM2	0.661	0.453	0.965	0.032
IGF1R	1.584	1.142	2.196	0.006
MUC1	1.457	1.106	1.921	0.008
PTGIS	1.500	1.069	2.105	0.019
ITGAM	0.424	0.233	0.773	0.005
KRAS	0.446	0.204	0.976	0.043
MSR1	0.604	0.370	0.986	0.044
PTPN3	1.973	1.076	3.618	0.028

Supplementary Table 2. OS-related gene expression set obtained after lasso regression.

id	TXN2	MAP3K5	G6PD	HMOX1	ATF4	CAT	ACADVL	MAPK1	INS	EIF2AK3	CCL2	MAPK10
1	6.12	4.11	4.93	7.20	8.54	6.11	6.66	6.40	2.77	5.52	4.87	3.68
2	6.39	3.84	5.51	8.24	9.14	5.30	8.13	5.80	2.77	4.42	7.48	3.08
3	6.13	3.91	5.62	6.75	7.95	6.30	8.47	6.07	2.77	5.04	4.67	2.97
4	7.13	3.79	4.87	4.69	8.43	5.59	7.85	6.38	2.77	5.42	3.31	3.33
5	6.22	3.11	5.22	4.15	8.38	6.77	6.60	6.09	2.77	4.67	3.83	3.08
6	6.17	3.54	5.45	5.70	8.09	5.48	7.28	5.60	2.77	5.72	3.22	2.99
7	6.60	4.56	4.66	5.80	9.94	5.39	7.16	6.20	2.87	5.32	2.99	2.90
8	5.57	3.41	5.60	5.31	7.59	5.62	6.70	6.54	2.77	5.58	3.19	3.42
9	6.47	3.79	5.73	6.75	9.30	5.52	9.03	5.67	2.77	4.94	5.04	2.97
10	6.92	4.82	5.48	9.44	8.81	6.08	7.95	6.24	2.77	4.47	4.14	3.75
11	6.91	3.92	4.88	6.29	9.37	6.12	7.97	7.25	2.77	5.17	4.75	2.97
12	5.15	3.52	5.37	5.95	9.93	4.88	7.78	6.36	2.77	5.22	2.84	2.91
13	6.51	3.54	5.05	7.92	8.49	6.56	7.71	7.11	2.77	5.35	4.34	2.94
14	6.62	3.28	4.94	5.97	9.20	5.39	8.80	6.77	2.77	5.87	3.39	2.98
15	6.48	3.28	4.48	6.40	9.62	4.86	7.51	7.05	2.77	5.63	3.07	4.60
16	6.99	4.34	5.37	9.99	9.27	5.76	8.37	6.14	2.77	4.82	6.01	3.17
17	5.83	3.81	5.73	4.86	7.75	5.75	6.86	6.13	2.77	5.14	3.06	3.03
18	6.48	5.00	5.72	6.00	8.07	6.40	7.47	6.32	2.77	4.76	6.43	2.96
19	6.49	3.75	5.17	6.96	9.58	5.84	8.15	6.93	2.76	5.12	3.55	3.18
20	7.92	6.01	4.68	6.24	9.72	6.36	7.71	7.67	2.77	4.93	3.47	2.99
21	6.47	4.71	5.20	10.35	8.05	7.15	7.81	6.58	2.77	4.55	7.25	2.94
22	7.25	3.87	5.76	7.05	8.31	5.74	7.23	5.81	2.77	4.25	6.28	3.24
23	6.55	3.90	5.05	6.71	8.39	6.10	7.51	6.29	2.76	4.70	4.15	3.99
24	5.92	3.74	5.13	7.53	8.44	5.84	7.31	6.62	2.77	5.48	4.74	2.95
25	6.32	3.96	5.44	6.59	9.22	6.27	7.22	6.79	2.77	5.77	4.74	2.95
26	6.60	3.92	4.86	6.00	8.59	6.34	6.78	6.44	2.77	4.83	4.09	3.15
27	6.43	4.58	5.64	9.58	8.13	6.36	7.07	6.70	2.77	4.33	5.03	3.65
28	7.04	3.69	5.02	5.84	10.56	5.85	6.84	6.66	2.76	3.70	3.42	3.58
29	6.78	4.20	6.28	6.99	9.23	5.58	7.75	6.28	2.76	4.64	5.39	4.12
30	7.23	3.39	5.79	6.31	9.64	6.04	8.40	6.77	2.78	5.54	3.66	3.86
31	5.59	3.65	4.90	8.92	9.08	5.90	7.26	6.35	2.77	5.13	4.33	3.42
32	6.91	4.14	5.81	7.61	8.36	5.98	7.67	7.17	2.77	5.57	3.52	3.08
33	6.75	4.32	5.33	6.56	9.29	5.38	7.73	6.07	2.77	5.14	5.03	3.18
34	6.06	3.34	5.13	6.11	9.32	4.67	7.51	5.84	2.77	5.93	3.21	2.93
35	6.50	3.57	5.16	6.23	8.58	5.80	7.50	5.66	2.76	5.06	4.39	2.93
36	6.72	5.07	6.89	8.91	8.60	6.39	7.21	6.22	2.77	5.06	5.46	3.35
37	7.12	3.41	5.21	6.22	9.34	6.58	7.30	6.13	2.77	3.98	3.86	3.88
38	6.70	4.47	4.51	5.19	8.83	4.71	7.77	5.53	2.76	4.76	3.23	3.00
39	6.38	3.37	5.40	4.78	8.76	5.20	8.02	6.41	2.77	4.76	3.11	3.38
40	6.84	4.08	5.56	9.65	9.51	5.54	7.76	6.45	2.77	4.66	7.13	3.37
41	6.66	3.75	5.81	6.89	8.45	6.41	7.31	6.06	2.77	4.70	4.88	2.97
42	6.60	4.16	5.75	8.96	8.21	5.87	7.92	6.04	2.77	4.79	6.27	3.10
43	7.26	4.04	6.49	8.30	9.29	6.08	7.92	6.06	2.76	4.67	5.12	3.49
44	6.16	3.97	5.68	6.13	7.67	5.84	6.87	6.06	2.77	4.48	4.43	3.34
45	6.29	3.63	5.36	8.31	7.82	5.58	7.25	6.80	2.77	4.65	4.15	3.43
46	5.87	3.97	5.46	6.90	9.09	6.81	8.08	6.05	2.76	5.27	3.75	2.97

47	6.68	3.94	5.11	7.01	9.32	6.25	6.82	6.51	2.77	5.18	4.21	2.93
48	7.04	4.16	5.04	6.34	9.20	6.07	7.70	6.61	2.77	4.79	4.38	3.39
49	7.04	4.43	5.81	6.63	8.56	5.86	7.11	6.93	2.77	5.40	5.07	3.60
50	6.78	3.15	5.54	6.68	8.48	6.05	8.23	6.18	2.77	5.13	4.74	2.97
51	6.72	4.13	5.61	5.31	9.40	5.55	6.19	6.18	2.77	4.81	4.41	3.01
52	6.74	3.83	5.19	7.17	9.58	5.71	7.98	6.03	2.77	5.38	4.36	3.00
53	7.47	4.28	5.43	5.97	8.71	5.49	8.13	5.28	2.77	3.50	3.59	4.08
54	6.95	3.74	6.29	8.93	9.02	5.82	7.66	6.21	2.77	5.09	3.97	3.00
55	7.60	4.31	6.01	6.81	10.15	5.50	7.47	6.37	2.76	5.00	4.75	4.29
56	6.55	3.78	4.95	6.18	8.77	5.48	6.57	7.02	2.77	5.22	4.53	3.63
57	5.48	4.03	5.39	5.10	8.00	5.67	7.72	6.00	2.77	5.09	3.99	3.08
58	6.37	3.67	5.84	5.73	8.85	6.01	7.48	6.81	2.77	4.03	4.90	3.18
59	6.32	3.64	6.14	9.41	8.44	6.10	7.63	6.33	2.77	4.13	5.72	3.03
60	7.77	5.23	7.33	6.85	9.68	5.34	7.65	5.80	2.77	3.69	7.09	3.09
61	6.23	3.11	5.73	5.53	8.62	6.07	6.96	7.81	2.77	4.48	3.68	4.80
62	7.21	3.67	6.63	7.03	8.33	5.43	7.35	5.39	2.77	3.37	5.21	3.81
63	7.33	4.24	5.79	6.87	8.36	5.90	6.92	6.39	2.77	4.21	7.27	2.93
64	6.95	3.56	6.01	7.80	9.42	5.95	7.80	6.27	2.77	4.99	5.73	3.23
65	6.49	3.80	4.71	6.65	8.74	5.80	7.78	6.08	2.77	4.83	3.92	2.94
66	6.80	4.21	5.47	5.21	7.83	6.43	7.17	5.48	2.77	4.68	4.77	2.95
67	6.20	5.08	5.28	6.50	8.69	4.82	6.93	6.47	2.77	5.26	4.74	2.96
68	6.89	3.57	5.67	8.12	9.34	4.93	7.95	5.56	2.77	6.16	4.27	3.11
69	6.15	5.14	6.63	6.07	8.53	5.94	8.59	6.52	2.77	5.66	4.33	3.20
70	7.44	4.88	5.21	6.24	9.27	6.29	8.12	6.59	2.76	5.63	5.68	3.24
71	5.73	3.32	4.42	8.36	8.82	5.89	6.94	5.72	2.77	5.23	5.05	3.23
72	6.76	3.26	5.69	8.65	8.33	5.69	7.71	6.66	2.77	4.68	4.66	3.03
73	6.76	3.80	7.09	8.26	9.01	6.10	8.18	6.30	2.77	5.31	5.48	2.98
74	7.12	4.02	5.79	8.12	9.69	4.22	7.62	6.41	2.77	4.72	6.94	3.16
75	6.70	4.43	6.36	8.24	8.77	5.63	7.50	6.06	2.77	4.56	6.13	3.34
76	7.19	3.80	6.18	8.38	8.53	5.39	7.44	6.13	2.77	4.59	5.36	3.08
77	6.92	3.58	5.96	7.89	10.56	5.28	8.08	6.48	2.76	5.67	3.77	3.29
78	6.88	3.85	6.99	9.26	9.08	6.03	7.17	6.94	2.76	5.02	6.37	3.33
79	6.16	4.46	5.90	6.58	8.27	6.79	7.66	6.97	2.77	4.73	4.42	3.06
80	6.70	4.83	5.34	7.89	8.77	6.54	8.53	6.66	2.76	4.32	6.84	3.04
81	6.93	3.76	4.97	6.30	9.48	6.64	7.78	6.34	2.77	5.12	5.96	2.99
82	6.32	3.41	4.82	6.75	8.94	5.66	7.95	6.12	2.77	4.62	5.12	2.90
83	6.50	4.25	5.37	5.46	9.00	6.13	6.99	6.07	2.77	5.19	7.73	3.09
84	6.68	4.29	6.19	6.19	8.26	6.09	6.51	6.36	2.77	4.32	4.18	3.00
85	6.97	4.17	5.47	9.51	8.98	6.02	8.15	6.29	2.76	4.75	4.77	2.95
86	6.88	5.08	5.57	7.06	8.15	5.19	7.69	6.90	2.76	3.93	5.34	3.06
